# EmPHasis-10 health-related quality of life score predicts outcomes in patients with idiopathic and connective tissue disease-associated pulmonary arterial hypertension: results from a UK multicentre study

**DOI:** 10.1183/13993003.00124-2020

**Published:** 2021-02-25

**Authors:** Robert A. Lewis, Iain Armstrong, Carmel Bergbaum, Melanie J. Brewis, John Cannon, Athanasios Charalampopoulos, A. Colin Church, J. Gerry Coghlan, Rachel J. Davies, Konstantinos Dimopoulos, Charlie Elliot, J. Simon R. Gibbs, Wendy Gin-Sing, Gulam Haji, Abdul G. Hameed, Luke S. Howard, Martin K. Johnson, Aleksander Kempny, David G. Kiely, Francesco Lo Giudice, Colm McCabe, Andrew J. Peacock, Oyinkansola Peleyeju, Joanna Pepke-Zaba, Gary Polwarth, Laura Price, Ian Sabroe, Benjamin E. Schreiber, Karen Sheares, Dolores Taboada, A.A. Roger Thompson, Mark R. Toshner, Ivy Wanjiku, S. John Wort, Janelle Yorke, Robin Condliffe

**Affiliations:** 1 Sheffield Pulmonary Vascular Disease Unit, Royal Hallamshire Hospital, Sheffield, UK; 2 Dept of Infection, Immunity and Cardiovascular Disease, University of Sheffield, Sheffield, UK; 3 National Pulmonary Hypertension Service, Royal Brompton Hospital and Imperial College, London, UK; 4 Scottish Pulmonary Vascular Unit, Golden Jubilee National Hospital, Glasgow, UK; 5 Pulmonary Vascular Disease Unit, Royal Papworth Hospital, Cambridge, UK; 6 Pulmonary Hypertension Unit, Royal Free Hospital, London, UK; 7 National Pulmonary Hypertension Service, Hammersmith Hospital, London, UK; 8 School of Nursing, Midwifery and Social Work, University of Manchester, Manchester, UK

## Abstract

Health-related quality of life (HRQoL) scores assess symptom burden in pulmonary arterial hypertension (PAH) but data regarding their role in prognostication and risk stratification are limited. We assessed these relationships using the emPHasis-10 HRQoL measure.

1745 patients with idiopathic PAH (IPAH), drug-induced PAH (DPAH), heritable PAH (HPAH) (collectively “(I/D/H)PAH”), or connective tissue disease-associated PAH (CTD-PAH), who had completed emPHasis-10 questionnaires at one of six UK referral centres between 2014 and 2017, were identified. Correlations with exercise capacity and World Health Organization (WHO) functional class were assessed, and exploratory risk stratification thresholds were tested.

Moderate correlations were seen between emPHasis-10 scores and 6-min walk distance (r=−0.546), incremental shuttle walk distance (r=−0.504) and WHO functional class (r=0.497) (all p<0.0001). Distribution of emPHasis-10 score differed significantly between each WHO functional class (all p<0.0001). On multivariate analysis, emPHasis-10 score, but not WHO functional class, was an independent predictor of mortality. In a risk stratification approach, scores of 0–16, 17–33 and 34–50 identified incident patients with 1-year mortality of 5%, 10% and 23%, respectively. Survival of patients in WHO functional class III could be further stratified using an emPHasis-10 score ≥34 (p<0.01). At follow-up, patients with improved emPHasis-10 scores had improved exercise capacity (p<0.0001) and patients who transitioned between risk groups demonstrated similar survival to patients originally in those risk groups.

The emPHasis-10 score is an independent prognostic marker in patients with (I/D/H)PAH or CTD-PAH. It has utility in risk stratification in addition to currently used parameters. Improvement in emPHasis-10 score is associated with improved exercise capacity.

## Abstract 2

**The emPHasis-10 health-related quality of life score is an independent predictor of mortality in idiopathic and connective tissue disease-related pulmonary arterial hypertension, and has utility in risk stratification.** https://bit.ly/2MrLFLn

## Introduction

Pulmonary arterial hypertension (PAH) is a rare condition, characterised by increased pulmonary vascular resistance (PVR) and progressive right-ventricular failure leading to premature death [1]. Exertional breathlessness and limitation in physical activity are typically the earliest reported symptoms and may be caused by a number of mechanisms [2, 3]. Exercise limitation may be objectively assessed by exercise testing, but limitations of day-to-day physical activity are typically assessed by healthcare professionals using the World Health Organisation (WHO) functional class system.

The importance of assessing patient-reported outcome measures (PROMs) in patients with pulmonary hypertension (PH) is now recognised [4, 5] and three PH-specific tools for assessing health-related quality of life (HRQoL) have been developed [6–8]. One of these tools, emPHasis-10, is comprised of 10 fields and results in a score out of 50 where a higher score represents a higher symptom burden. It can be completed quickly by patients and is free to use, and so is well-suited to routine clinical use [7]. The emPHasis-10 score was found to correlate strongly with measures of HRQoL, breathlessness and psychological morbidity, and has high test–retest and internal consistency [7]. In addition, the emPHasis-10 questionnaire has been translated into a number of other languages [9, 10]. A previous single-centre study of emPHasis-10 in patients with PAH (predominantly associated with congenital heart disease) and chronic thromboembolic pulmonary hypertension (CTEPH) demonstrated prognostic significance and a correlation with WHO functional class [11]. Although risk stratification has an established central role in the management of patients with PAH, PROMs are not incorporated in current risk assessment tools [12–14].

Routine HRQoL assessment using a PH-specific tool has been a mandatory field in the UK National Audit of Pulmonary Hypertension since 2014 [15]. We performed a multi-centre study on a large cohort of patients with idiopathic PAH (IPAH), drug-induced PAH (DPAH), heritable PAH (HPAH) (collectively “(I/D/H)PAH”) and connective tissue disease-associated PAH (CTD-PAH), to further assess the relationship between emPHasis-10 score and mortality, to identify correlations with clinical parameters (including exercise capacity) and to determine whether a threshold approach for risk stratification could be applied.

## Methods

Local databases for six out of the seven PH-referral centres in the UK, which together manage 94% of adult patients with a diagnosis of PAH, were interrogated [15]. Patients with PAH were diagnosed as per contemporaneous international guidelines (mean pulmonary arterial pressure (PAP) ≥25 mmHg and pulmonary arterial wedge pressure (PAWP) ≤15 mmHg in the absence of thromboembolic disease or conditions associated with other forms of PH) [16]. Anonymised demographic, haemodynamic, spirometric, exercise, emPHasis-10 and mortality data were retrieved for all patients with a diagnosis of IPAH, DPAH, HPAH (hereafter grouped as “(I/D/H)PAH”), or CTD-PAH with at least one recorded emPHasis-10 score between January 01, 2014 and May 31, 2018. Incident patients were required to have an emPHasis-10 score at the point of diagnosis, which was possible if diagnosed from 2014 onwards since its clinical use was introduced in the UK during that year. For prevalent patients (*i.e.* those diagnosed prior to 2014 or for whom no emPHasis-10 score was available at the time of diagnosis), the first available emPHasis-10 score was used. In either group, the first emPHasis-10 score was described as the baseline measurement. All patients were under regular clinical follow-up and the outcome measured was death or transplant by May 31, 2019. Follow-up data were retrieved for the first visit between 3 and 12 months after baseline emPHasis-10 score was measured.

### Statistical analysis

Statistical analysis was performed using SPSS Statistics version 26 (IBM Inc, Armonk, NY, USA) and GraphPad Prism version 8 (GraphPad Spftware, San Diego, CA, USA). Continuous data were displayed as either mean±standard deviation (sd) or median (1st–3rd quartile) for non-parametric data. Demographics were compared using paired and unpaired T-tests for parametric data and Wilcoxon signed-rank and Mann–Whitney U-tests for non-parametric data. Frequencies were compared using Chi-squared. For Cox regression modelling, parameters of known prognostic significance in PAH were utilised (age, gender, presence of CTD (rather than (I/D/H)PAH), mean right atrial pressure (RAP), cardiac index and walking distance. Collinearity was assessed by measuring the variance inflation factor and tolerance between variables. EmPHasis-10 score was entered as a continuous variable in the multivariable model. Multivariate Cox regression analysis was performed in a forward direction on all parameters with a p-value of less than 0.2 on univariate analysis. Data were scaled to the mean and hazard ratios were based on the z-score. Two types of walking test were used (the 6-min walk test (6MWT) and the incremental shuttle walk test (ISWT)) and so, for multivariate modelling, distances were converted to a z-score and combined as a single variable. For all statistical tests other than multivariate analysis, a p-value of less than 0.05 was considered significant. Kaplan–Meier survival curves were compared using log-rank Chi-squared and were truncated at 4 years based on the census date. Correlations were assessed using either Pearson or Spearman rank, as appropriate. Risk models were compared using the c-statistic identified from receiver operating characteristic (ROC) curve analysis. The minimal detectable change (MDC) for emPHasis-10 score was calculated using the formula: MDC=1.96·√2·standard error (se) of measurement [17]. Ethical approval was granted (IRAS 254446).

## Results

A total of 1745 patients with (I/D/H)PAH (n=994) or CTD-PAH (n=751) who had at least one recorded emPHasis-10 score were identified. There was a female predominance (73%) and 35% of patients were incident and treatment-naïve at the time of baseline emPHasis-10 score measurement. The emPHasis-10 score was higher in patients with CTD-PAH (median 30 (19–38)) than in patients with (I/D/H)PAH (median 28 (17–37)) (p=0.001). Baseline demographics are displayed in table 1.

**TABLE 1:** Patient characteristics

	**All (1745)**	**(I/D/H)PAH (n=994)**	**CTD-PAH (n=751)**	**p-value**
**Female sex %**	73	66	82	<0.0001
**Age at diagnosis years**	59±17	55±18	64±13	<0.0001
**Incident patients % (n=618)**	35	29	44	<0.0001
**FEV_1_ % predicted (n=1457)**	82±21	84±19	80±23	0.0001
**FVC % predicted (n=1459)**	92±23	95±20	89±27	<0.0001
**FEV_1_/FVC (n=1459)**	73±13	74±13	73±14	0.078
**Mean RAP mmHg (n=1503)**	9±6	10±6	8±5	<0.0001
**Mean PAP mmHg(n=1573)**	48±13	53±13	41±11	<0.0001
**PAWP mmHg (n=1496)**	9±4	9±4	9±3	0.56
**PVR Wood units (n=1378)**	10.5±5.8	12.0±5.7	8.7±5.4	<0.0001
**Cardiac output L·min^−1^ (n=1465)**	4.2±1.5	4.0±1.5	4.3±1.5	<0.0005
**Cardiac index L·min^−1^·m^−2^ (n=1305)**	2.4±0.8	2.2±0.8	2.5±0.8	<0.0001
**EmPHasis-10 score**	29 (18–38)	28 (17–37)	30 (19–38)	0.001
**WHO functional class^#^ % (n=1725)**				
Class I	3	4	1	
Class II	23	26	20	
Class III	61	57	67	
Class IV	13	13	12	
**6MWD^#^ (n=659)**	310 (180–408)	340 (192–432)	241 (141–360)	<0.0001
**ISWD^#^ (n=797)**	150 (70–270)	160 (80–350)	140 (60–228)	0.001

Data are presented as n, mean±sd, or median (1st–3rd quartile). PAH: pulmonary arterial hypertension; CTD: connective tissue disease; IPAH: idiopathic PAH; DPAH: drug-induced PAH; HPAH: heritable PAH; CTD-PAH: CTD-associated PAH; FEV_1_: forced expiratory volume in 1 s; FVC: forced vital capacity; RAP: right atrial pressure; PAP: pulmonary arterial pressure; PAWP: pulmonary arterial wedge pressure; PVR: pulmonary vascular resistance; WHO: World Health Organization; 6MWD: 6-min walk distance; ISWD: incremental shuttle walk distance; sd: standard deviation. ^#^: variables were recorded at the time of baseline emPHasis-10 measurement (other variables were recorded at diagnosis). Baseline 6MWD and ISWD were available in 38% and 46% of patients, respectively, with no overlap.

### Correlation with clinical parameters

Moderate correlations (all p<0.0001) were seen between baseline emPHasis-10 score and WHO functional class (r=0.50), 6-min walk distance (6MWD) (r=−0.55) and incremental shuttle walk distance (ISWD) (r=−0.50), as illustrated in table 2. In incident patients with right-heart catheter data available (n=591), there were weak correlations with mean RAP (r=0.21), cardiac index (r=−0.21) and PVR (r=0.17) (all p<0.0001). Correlations were similar in the subgroups of (I/D/H)PAH and CTD-PAH, apart from PVR where correlation was significant in CTD-PAH (r=0.21; p<0.0005) but not in (I/D/H)PAH (r=0.11; p=0.8). Correlations between WHO functional class, walk distance and haemodynamics are also shown in table 2.

**TABLE 2:** Correlation of emPHasis-10 score and World Health Organization (WHO) functional class with walk distance and pulmonary haemodynamics

	**6MWD m**	**ISWD m**	**Mean RAP mmHg**	**Cardiac index L·min**^−1^**·m**^−**2**^	**PVR Wood units**
**EmPHasis-10 score**	−0.55^#^ (n=659)	−0.50^#^ (n=797)	0.21^#^ (n=575)	−0.21^#^ (n=525)	0.17^#^ (n=550)
**WHO functional class**	−0.60^#^ (n=653)	−0.59^#^ (n=796)	0.18^#^ (n=572)	−0.18^#^ (n=523)	0.18^#^ (n=548)

Correlation was assessed by Pearson or Spearman-rank tests as appropriate. 6MWD: 6-min walk distance; ISWD: incremental shuttle walk distance; RAP: right atrial pressure; PVR: pulmonary vascular resistance. ^#^: p<0.001.

**FIGURE 1:**
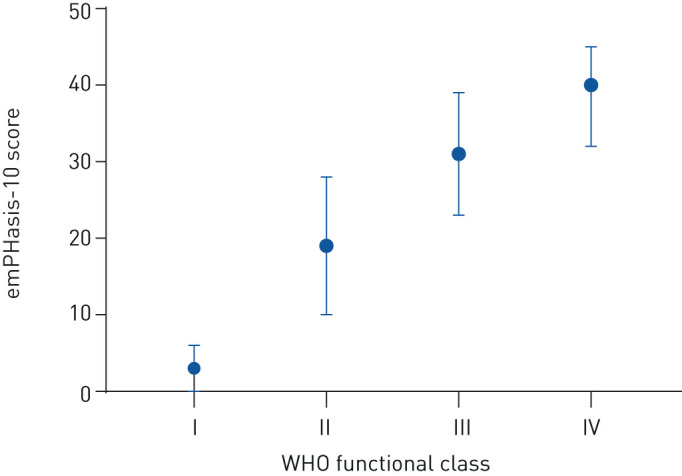
Distribution of emPHasis-10 score by World Health Organization (WHO) functional class at baseline

Distribution of emPHasis-10 score by WHO functional class at baseline is shown in figure 1. Median emPHasis-10 scores were 3, 19, 31 and 40 in WHO functional classes I, II, III and IV, respectively, with highly significant differences between the scores in each functional class (all p<0.0001).

### Risk stratification

**FIGURE 2:**
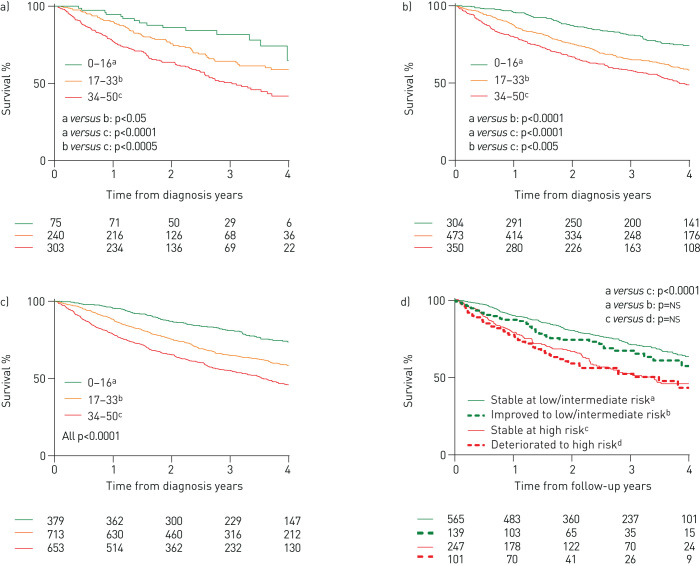
Kaplan–Meier survival curves demonstrating survival from baseline emPHasis-10 score for a) incident patients, b) prevalent patients and c) all patients, as well as d) risk transition in all patients between baseline and follow-up emPHasis-10 score. ns: not significant.

**FIGURE 3:**
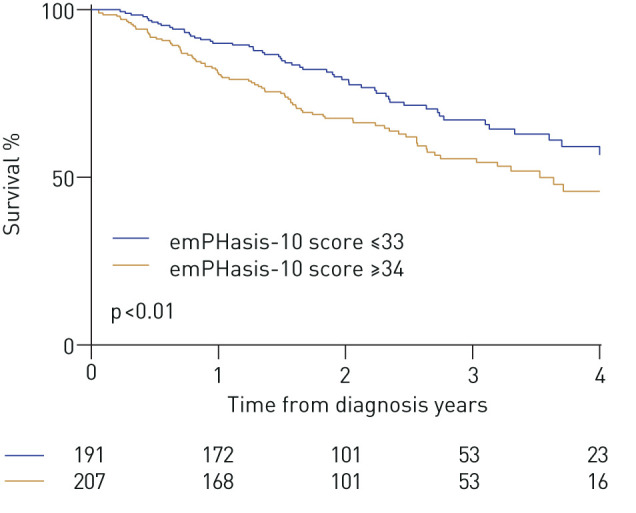
Survival in incident patients with World Health Organization (WHO) functional class III symptoms, stratified by emPHasis-10 score (≤33 or ≥34).

During the course of the study 674 patients (39%) died, of which 240 (14%) died within 1 year of baseline emPHasis-10 score measurement. The 1-year mortality in incident and prevalent patients was 16% and 12%, respectively. An exploratory three-level score was developed based on a tertile group approach: scores of 0–16 were defined as low-risk, 17–33 as intermediate-risk and 34–50 as high-risk. Using these thresholds, 22% of all patients were defined as low-risk for 1-year mortality, 41% as intermediate-risk and 37% as high-risk. Survival curves for these risk groups are shown for incident patients in figure 2a, for prevalent patients in figure 2b and for all patients in figure 2c. In incident patients, 1-year mortality for the low, intermediate and high-risk groups was 5%, 10% and 23%, respectively. In prevalent patients it was 4%, 13% and 20%, respectively, while for all patients it was 4%, 12% and 21%, respectively. In all patients with (I/D/H)PAH, 1-year mortality for the low, intermediate and high-risk groups was 4%, 9% and 18%, respectively, whereas in CTD-PAH it was 6%, 15% and 25%. Incident patients in functional class III who were in the low/intermediate emPHasis-10 risk group (emPHasis-10 score 0–33) had superior survival than those in the high-risk group (emPHasis-10 score 34–50), with 1-year survival of 90% *versus* 81% and 3-year survival of 67% *versus* 56% (p<0.01) (figure 3). Very similar observations were made in functional class III patients at their first follow-up visit.

### Survival analysis

Three multivariate analysis models were developed in the incident population (table 3). Model 1 utilised accepted prognostic parameters (age, gender, CTD-PAH rather than (I/D/H)PAH, WHO functional class, mean RAP and cardiac index). EmPHasis-10 and exercise capacity were sequentially added into models 2 and 3. Unlike WHO functional class, emPHasis-10 score was an independent predictor of outcome in model 2 (scaled hazard ratio 1.565, p<0.0001) and model 3 (scaled hazard ratio 1.226, p<0.05). There was no significant collinearity between parameters used in the model.

**TABLE 3:** Univariate and multivariate analysis in incident patients

**Model 1**	**Univariate**		**Multivariate**	
	**Scaled hazard ratio**	**p-value**	**Scaled hazard ratio**	**p-value**
**Model 1**				
Age	2.063	<0.0001	2.177	<0.0001
Gender (ref. female)	1.316	0.054		
CTD-PAH (ref. (I/D/H)PAH)	1.336	0.031	1.444	0.017
WHO functional class III (ref. class I and II)	1.817	0.009		
WHO functional class IV (ref. class I and II)	3.642	<0.0001	2.978	<0.0001
Mean RAP	1.196	0.006	1.227	0.005
Cardiac index	0.756	0.001		
**Model 2**				
Age	2.063	<0.0001	2.180	<0.0001
Gender (ref. female)	1.316	0.054		
CTD-PAH (ref. (I/D/H)PAH)	1.336	0.031		
WHO functional class III (ref. class I and II)	1.817	0.009		
WHO functional class IV (ref. class I and II)	3.642	<0.0001		
Mean RAP	1.196	0.006		
Cardiac index	0.756	0.001		
EmPHasis-10 score	1.518	<0.0001	1.447	<0.0001
**Model 3**				
Age	2.063	<0.0001	1.860	<0.0001
Gender (ref. female)	1.316	0.054		
CTD-PAH (ref. (I/D/H)PAH)	1.336	0.031		
WHO functional class III (ref. class I and II)	1.817	0.009		
WHO functional class IV (ref. class I and II)	3.642	<0.0001		
Mean RAP	1.196	0.006		
Cardiac index	0.756	0.001		
EmPHasis-10 score	1.518	<0.0001	1.226	0.047
Walk distance^#^	0.461	<0.0001	0.574	<0.0001

PAH: pulmonary arterial hypertension; CTD: connective tissue disease; IPAH: idiopathic PAH; DPAH: drug-induced PAH; HPAH: heritable PAH; CTD-PAH: CTD-associated PAH; WHO: World Health Organization; RAP: right-atrial pressure. ^#^: two types of walking test were used, the 6-min walk test (6MWT) and the incremental shuttle walk test (ISWT). For Cox regression modelling, distances were converted to a z-score and combined.

### Magnitude of change

The MDC for emPHasis-10 score was calculated to be nine. Follow-up emPHasis-10 data were available for 1068 patients (61%). EmPHasis-10 score changed by at least nine points between baseline and follow-up in 33% of patients (32% for (I/D/H)PAH and 34% for CTD-PAH). Thirty-seven percent of patients moved risk groups, of which 19% improved by at least one risk group. In patients who moved from high risk to intermediate or low risk, the median change in emPHasis-10 score was −12 points (−6 to −19 points) and in patients who deteriorated to high-risk the median change was +13 points (+8 to +17 points). Patients who either improved to low or intermediate-risk, or deteriorated to high-risk, demonstrated similar long-term survival to patients originally in those risk groups (figure 2d).

**FIGURE 4:**
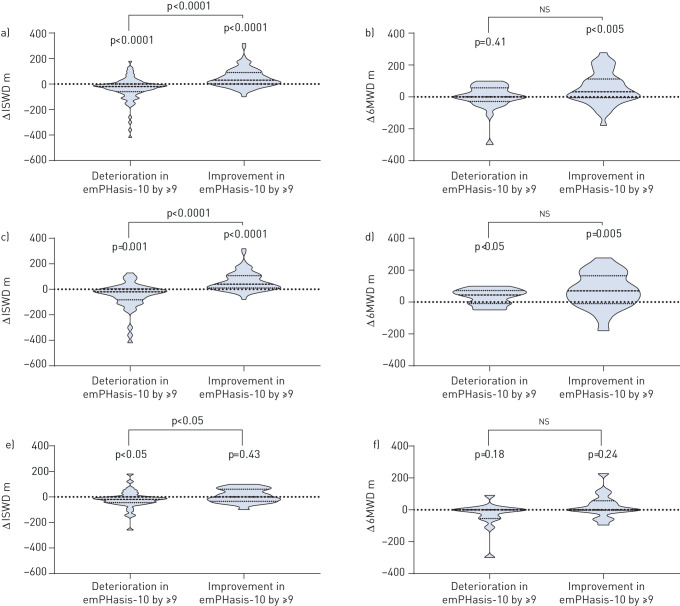
Change, as illustrated by violin plots (where: dashed line=median; dotted lines=25th and 75th percentiles), in incremental shuttle walk distance (ISWD) and 6-min walk distance (6MWD) in patients whose emPHasis-10 score deteriorated by ≥9 or improved by ≥9 between baseline and follow-up ((a and b) all patients, (c and d) incident patients and (e and f) prevalent patients).

On paired testing in patients with a follow-up emPHasis-10 score, those who improved emPHasis-10 score by the MDC of nine points or greater had significantly improved walk distances at follow-up. ISWD increased by 30 m (0–90 m) (p<0.0001) (figure 4a) while 6MWD increased by a median distance of 32 m (−4 to 113 m) (p<0.005) (figure 4b). A significant fall in ISWD of −20 m (−60 to 0 m) (p<0.0001) (figure 4a) and no significant change in 6MWD (0 m, −29 to 57 m) (figure 4b) was observed in patients whose emPHasis-10 score deteriorated by at least nine points. In the remaining patients, in whom there was a change of less than nine points, there was no significant change in either ISWD (0 m, −30 to 20 m) or 6MWD (0 m, −11 to 53 m). The relationship between the change in emPHasis-10 score and the change in walk distance differed depending on whether patients were incident or prevalent at the time of their baseline walk (the relationship being stronger in incident patients) and whether they performed the ISWT or 6MWT (the relationship being stronger in patients who performed the ISWT). In patients whose emPHasis-10 score deteriorated by nine or more points, ISWD fell significantly in both the incident and prevalent populations. In patients whose emPHasis-10 score improved by nine or more points, ISWD increased significantly in incident but not prevalent, patients (figures 4c and 4e). In patients for whom a 6MWT was performed, an improvement was observed in incident patients whose emPHasis-10 score either improved or deteriorated by nine or more points (figure 4d), while no significant change was seen in prevalent patients (figure 4f).

## Discussion

To our knowledge, this is the largest study to assess the role of quality of life (QoL) scores in patients with PAH. In this multi-centre study we report on data from centres treating the vast majority of the adult PAH population in the UK. We have demonstrated that the emPHasis-10 score is an independent predictor of outcomes when adjusting for haemodynamics and WHO functional class, and also that it has utility in risk stratification, including within patients in WHO functional class III. We have also observed moderate correlations with WHO functional class and exercise capacity, and weaker correlations with pulmonary haemodynamics. Furthermore, we have also demonstrated that improvement in emPHasis-10 score, as opposed to a static or worsening score, is associated with improvements in exercise capacity.

Generic (*e.g.* the 36-item Short Form Health Survey (SF-36)), heart-failure specific (*e.g.* the Minnesota Living with Heart Failure Questionnaire (MLHFQ)) and PH-specific (*e.g.* emPHasis-10 and the Cambridge Pulmonary Hypertension Outcome Review (CAMPHOR)) PROMs have previously been identified as having prognostic importance in PAH [11, 18–20]. Correlations between CAMPHOR and both SF-36 and 6MWD have also been demonstrated [19, 21, 22]. The widespread clinical use of the CAMPHOR score may, however, be limited by its length (65 fields over three domains: symptoms, functioning and QoL) and lack of open access [23]. A third PH-specific PROM, the Pulmonary Arterial Hypertension-Symptoms and Impact (PAH-SYMPACT) tool, which consists of 22 fields over two domains: symptoms and impacts, has also been developed [8]. Although PAH-SYMPACT is responsive to change, its relationship to haemodynamics and survival is not known [24].

A previous single-centre study involving 687 patients (congenital heart disease-associated PAH (n=314), (I/D/H)PAH (n=109), CTD-PAH (n=111) and CTEPH (n=131)) assessed the relationship between emPHasis-10 and survival [11]. In that study, Cox regression analysis demonstrated emPHasis-10 to be predictive of survival, independent of WHO functional class, in congenital heart disease-associated PAH, but not in (I/D/H)PAH or CTD-PAH. In our study, which included much larger numbers of patients with (I/D/H)PAH and CTD-PAH, emPHasis-10 was an independent prognostic marker in (I/D/H)PAH and CTD-PAH, even when allowing for a number of variables known to be strongly prognostic in PAH (including mean RAP and cardiac index). This was not the case for WHO functional class and it is interesting to note that Boucly *et al.* [14] also observed that baseline WHO functional class was not an independent predictor of outcome in their paper from the French Registry.

In incident patients, an exploratory risk stratification approach separating emPHasis-10 scores into three bands, based on an equal range of scores in each group (thresholds of ≤16, 17–33 and ≥34), identified distinct risk groups with significant survival differences (corresponding 1-year mortality of 5%, 10% and 23%, respectively). These levels of 1-year mortality are very similar to the risk thresholds of low (<5%), intermediate (5–10%) and high (>10%) risk proposed by the European Society of Cardiology (ESC)/European Respiratory Society (ERS) guidelines for risk stratification in PAH. Using these risk thresholds, we determined that patients who either improved to intermediate or low risk at follow-up emPHasis-10 assessment, or deteriorated to high risk, had similar longer-term survival to those patients who were originally in those risk groups. This effect has been seen in a number of other risk stratification parameters and scores [25–27], and the importance of achieving specific PROM thresholds in PAH has also been observed with the generic SF-36 [28]. The majority of patients were in WHO functional class III at the time of diagnosis and we were therefore interested in whether the emPHasis-10 score could refine these patients into higher and lower risk groups. We observed that a threshold score ≥34 was indeed able to identify WHO functional class III patients at higher and lower risk of 1-year mortality at both diagnosis and at first follow-up.

We observed moderate correlations between emPHasis-10 and exercise capacity (6MWD, r=0.55; ISWD, r=0.50) and between emPHasis-10 and WHO functional class (r=0.50), and only weak correlations with pulmonary haemodynamics (r=0.17–0.21). The correlations between emPHasis-10 and exercise capacity compare favourably with some reports regarding the other two PH-specific PROMs. Gomberg-Maitland *et al.* [22] reported weaker correlations between 6MWD and the three domains of CAMPHOR (symptoms (r=0.35), functioning (r=0.45) and QoL (r=0.33)) in 147 PAH patients, while Chin *et al.* [24] observed weak to moderate correlation between domains of the PAH-SYMPACT tool and 6MWD (r=−0.14 to −0.57) in 278 PAH patients. More recently however, Reis *et al.* [29] observed stronger correlation between 6MWD and the three CAMPHOR domains (symptoms (r=−0.67), functioning (r=−0.74) and QoL (r=−0.61)) in 49 patients with PAH or CTEPH. To date, there have been no reports of correlation of PH-specific PROMs with pulmonary haemodynamics. The correlations we observed were, however, comparable to those observed by Mathai *et al.* [18] between components of the generic SF-36 HRQoL tool and haemodynamics in 87 patients with PAH (although, in their study, many of these correlations were non-significant).

Finally, we have demonstrated that an improvement in emPHasis-10 score at follow-up of at least the MDC (nine or greater) was associated with an increase in exercise capacity in incident patients, whereas a reduction in emPHasis-10 score by nine or greater was associated with a decrease in exercise capacity when assessed by the ISWD. The vast majority of incident patients will have been started on PAH therapies, whereas in prevalent patients there may have been no treatment change between assessments, which may partly explain the stronger relationship between change in walk distance and change in emPHasis-10 score in the incident group. The reason for the stronger relationship between change in ISWD (as opposed to 6MWD) and change in emPHasis-10 is not clear, but may reflect the different nature of the tests (the ISWT is an externally-paced measure of maximal exercise capacity while the 6MWT is an internally-paced assessment of sub-maximal exercise capacity). These data suggest that emPHasis-10 is responsive to change; however, further work is needed to define the minimal clinically important difference.

### Limitations

While this study was able to demonstrate important associations between emPHasis-10 score and time to death or transplantation, other measures of clinical deterioration including hospitalisation due to heart failure and escalation of therapy were unavailable. In addition, while emPHasis-10 scores were prospectively collected, this was a retrospective study and there were some data availability issues. Treatment data were not available and it is possible that PAH-specific therapies might affect HRQoL both negatively (in terms of side effects and the effects of complex treatments on lifestyle) and also positively (in terms of improvements in right-ventricular function translating into amelioration of symptoms). Finally, data regarding comorbidities, such as the presence and extent of parenchymal lung disease in patients with CTD-PAH, were unavailable. Assuming that comorbidities such as lung disease adversely affect HRQoL, the inclusion of patients with parenchymal lung disease would likely weaken the relationships between emPHasis-10 and functional parameters, treatment response and survival.

### Conclusion

The emPHasis-10 score correlates with WHO functional class, exercise capacity and haemodynamics, and is an independent prognostic marker in patients with (I/D/H)PAH and CTD-PAH. It has utility in risk stratification in addition to currently used parameters. The survival of patients within WHO functional class III can be further stratified using emPHasis-10 score. Improvement in emPHasis-10 is associated with improvement in exercise capacity, although further work to determine the minimal clinically important difference is required.

## Shareable PDF

10.1183/13993003.00124-2020.Shareable1 This one-page PDF can be shared freely online.
Shareable PDF ERJ-00124-2020.Shareable
