# Supplementary material for: EmPHasis-10 health-related quality of life score predicts outcomes in patients with idiopathic and connective tissue disease-associated pulmonary arterial hypertension: results from a UK multicentre study
Source: Eur Respir J. 2021 Feb 25;57(2):2000124. doi: 10.1183/13993003.00124-2020 (PMC7905834; doi:10.1183/13993003.00124-2020)
Supplement: Supplementary file 1 [file ERJ-00124-2020.Shareable.pdf]

SHAREABLE PDF

EmPHasis-10 health-related quality of life score predicts outcomes in patients with idiopathic and connective tissue disease-associated pulmonary arterial hypertension: results from a UK multicentre study

Robert A. Lewis[1,2], Iain Armstrong[1], Carmel Bergbaum[3], Melanie J. Brewis[4], John Cannon[5], Athanasios Charalampopoulos[1], A. Colin Church[4], J. Gerry Coghlan[6], Rachel J. Davies[7], Konstantinos Dimopoulos[3], Charlie Elliot[1], J. Simon R. Gibbs[7], Wendy Gin-Sing[7], Gulam Haji[7], Abdul G. Hameed[1,2], Luke S. Howard[7], Martin K. Johnson[4], Aleksander Kempny[3], David G. Kiely[1,2], Francesco Lo Giudice[7], Colm McCabe[3], Andrew J. Peacock[4], Oyinkansola Peleyeju[6], Joanna Pepke-Zaba[5], Gary Polwarth[5], Laura Price[3], Ian Sabroe[1,2], Benjamin E. Schreiber[6], Karen Sheares[5], Dolores Taboada[5], A.A. Roger Thompson[1,2], Mark R. Toshner[5], Ivy Wanjiku[6], S. John Wort[3], Janelle Yorke[8] and Robin Condliffe[1]

Affiliations: [1]Sheffield Pulmonary Vascular Disease Unit, Royal Hallamshire Hospital, Sheffield, UK. [2]Dept of Infection, Immunity and Cardiovascular Disease, University of Sheffield, Sheffield, UK. [3]National Pulmonary Hypertension Service, Royal Brompton Hospital and Imperial College, London, UK. [4]Scottish Pulmonary Vascular Unit, Golden Jubilee National Hospital, Glasgow, UK. [5]Pulmonary Vascular Disease Unit, Royal Papworth Hospital, Cambridge, UK. [6]Pulmonary Hypertension Unit, Royal Free Hospital, London, UK. [7]National Pulmonary Hypertension Service, Hammersmith Hospital, London, UK. [8]School of Nursing, Midwifery and Social Work, University of Manchester, Manchester, UK.

Correspondence: Robin Condliffe, Sheffield Pulmonary Vascular Disease Unit, Royal Hallamshire Hospital, Sheffield S10 2JF, UK. E-mail: robin.condliffe@nhs.net

@ERSpublications
The emPHasis-10 health-related quality of life score is an independent predictor of mortality in idiopathic and connective tissue disease-related pulmonary arterial hypertension, and has utility in risk stratification. https://bit.ly/2MrLFLn

Cite this article as: Lewis RA, Armstrong I, Bergbaum C, *et al.* EmPHasis-10 health-related quality of life score predicts outcomes in patients with idiopathic and connective tissue disease-associated pulmonary arterial hypertension: results from a UK multicentre study. *Eur Respir J* 2021; 57: 2000124 [https://doi.org/10.1183/13993003.00124-2020].

This single-page version can be shared freely online.

ABSTRACT Health-related quality of life (HRQoL) scores assess symptom burden in pulmonary arterial hypertension (PAH) but data regarding their role in prognostication and risk stratification are limited. We assessed these relationships using the emPHasis-10 HRQoL measure.

1745 patients with idiopathic PAH (IPAH), drug-induced PAH (DPAH), heritable PAH (HPAH) (collectively "(I/D/H)PAH"), or connective tissue disease-associated PAH (CTD-PAH), who had completed emPHasis-10 questionnaires at one of six UK referral centres between 2014 and 2017, were identified. Correlations with exercise capacity and World Health Organization (WHO) functional class were assessed, and exploratory risk stratification thresholds were tested.

Moderate correlations were seen between emPHasis-10 scores and 6-min walk distance (r=−0.546), incremental shuttle walk distance (r=−0.504) and WHO functional class (r=0.497) (all p<0.0001). Distribution of emPHasis-10 score differed significantly between each WHO functional class (all p<0.0001).

Copyright ©ERS 2021. This article is open access and distributed under the terms of the Creative Commons Attribution Licence 4.0.

On multivariate analysis, emPHasis-10 score, but not WHO functional class, was an independent predictor of mortality. In a risk stratification approach, scores of 0–16, 17–33 and 34–50 identified incident patients with 1-year mortality of 5%, 10% and 23%, respectively. Survival of patients in WHO functional class III could be further stratified using an emPHasis-10 score ⩾34 ($p<0.01$). At follow-up, patients with improved emPHasis-10 scores had improved exercise capacity ($p<0.0001$) and patients who transitioned between risk groups demonstrated similar survival to patients originally in those risk groups.

The emPHasis-10 score is an independent prognostic marker in patients with (I/D/H)PAH or CTD-PAH. It has utility in risk stratification in addition to currently used parameters. Improvement in emPHasis-10 score is associated with improved exercise capacity.
